# Optimizing Extracellular Products from *Vibrio proteolyticus* for Their Use as Postbiotics in Aquaculture

**DOI:** 10.1007/s10126-025-10500-6

**Published:** 2025-08-02

**Authors:** Jorge García-Márquez, Marta Domínguez-Maqueda, Olivia Pérez-Gómez, Isabel M. Cerezo, Cristóbal Espinosa-Ruíz, M. Ángeles Esteban, Fernando Vallejo, Francisco Javier Alarcón-López, Eduardo Martínez-Manzanares, Silvana Teresa Tapia-Paniagua, María Carmen Balebona, Miguel Ángel Moriñigo, Salvador Arijo

**Affiliations:** 1 Departamento de Microbiología, Facultad de Ciencias, Universidad de Málaga, Instituto Andaluz de Biotecnología y Desarrollo Azul (IBYDA), Campus Universitario de Teatinos S/N, 29071 Málaga, Spain; 2 Departamento de Biología Celular e Histología, Facultad de Ciencias, Universidad de Murcia, 30100 Murcia, Spain; 3 Centro de Edafología y Biología Aplicada del Segura (CEBAS-CSIC), Metabolomic Platform, Campus Universitario de Espinardo, 30100 Murcia, Spain; 4 Departamento de Biología y Geología, Universidad de Almería, Ceimar-Universidad de Almería, 04120 Almería, Spain; 5 LifeBioencapsulation SL. El Alquián, 04131 Almería, Spain

**Keywords:** Extracellular products, Microbial culture media, Postbiotic, Probiotic, *Vibrio proteolyticus*

## Abstract

*Vibrio* *proteolyticus* DCF12.2 has demonstrated its ability to be used as a probiotic for fish species. This study investigates how different culture conditions influence the activity of its extracellular products (ECPs) in aquaculture, focusing on enzymatic and antibacterial activity, cytotoxicity, biofilm modulation, short-chain fatty acid (SCFA) profiles, and effects on *Photobacterium damselae* subsp. *piscicida* virulence. Enzymatic assays showed a variety of hydrolytic activities, including amylase, caseinase, and collagenase, which can enhance digestion and nutrient absorption in fish. Antibacterial assays revealed that ECPs from *V. proteolyticus* grown in an experimental aquafeed and a partial replacement of that aquafeed by 25% of a blend of microalgae inhibited *P. damselae* subsp. *piscicida* and *P. damselae* subsp. *damselae*. Cytotoxicity assays indicated variable effects across fish cell lines, with increased viability in SAF-1 and DLB-1 cells under specific conditions, and decreased viability in PLHC-1 cells, suggesting potential antitumor properties. Biofilm assays showed that certain ECP conditions reduced biofilm formation by *Vibrio anguillarum*, *Aeromonas hydrophila*, and *Tenacibaculum maritimum*. SCFA profiling detected acetic, iso-valeric, butyric, and valeric acids, which may contribute to antimicrobial activity and gut health. The ECPs significantly downregulated *aip56* gene transcription, reducing the virulence of *P. damselae* subsp*. piscicida*. These findings suggest that ECPs from *V. proteolyticus* could be valuable aquafeed additives for enhancing fish nutrition, health, and disease resistance. Future research should aim to isolate and characterize the specific bioactive compounds responsible for these effects and elucidate their mechanisms of action for optimized application in aquaculture and other biotechnological fields.

**Supplementary Information:**

The online version contains supplementary material available at 10.1007/s10126-025-10500-6.

## Introduction

With the rising demand for aquatic animals, aquaculture practices have shifted from extensive to intensive culture systems, resulting in greater stressors such as overpopulation, frequent handling, transportation, and compromised water quality (Ciji and Akhtar 2021). This shift has led to a significant increase in disease occurrences, particularly within intensive and highly intensive culture environments. The widespread use of antibiotics to combat infectious diseases in these systems has raised significant concerns regarding the emergence and spread of antibiotic-resistant pathogens, which pose substantial risks to public health (Salam et al. 2023). In light of these limitations and the growing concerns over antibiotic use, alternative functional ingredients—including probiotics, prebiotics, bioactive compounds, polysaccharides, and medicinal herbs—have gained attention as potential substitutes for antibiotics and are increasingly being incorporated into aquafeeds.

Probiotics, defined as live microorganisms that confer health benefits when administered in adequate amounts (Hill et al. 2014), have been extensively studied for their potential to enhance human and farm animal health (Dawood et al. 2019; Tegegne and Kebede 2022). Despite their promising benefits, several concerns have been raised about their functionality and practical application. They include the probiotic viability in products or feed, their colonization patterns and persistence in the gut, and the potential for horizontal gene transfer of virulence genes from pathogenic bacteria (Newaj-Fyzul et al. 2014).

In response to these concerns, alternative approaches, such as the use of postbiotics, have emerged. Postbiotics are non-viable bacterial products or metabolic by-products, including bacteriocins, organic acids, extracellular products, and enzymes, that exhibit beneficial biological activity in the host (Aguilar-Toalá et al. 2018). The understanding of postbiotics and their impact on host health has significantly advanced in recent years (Moradi et al. 2021), making them a compelling alternative to biological approaches to disease control (Barros et al. 2020). Postbiotics demonstrate promising properties, such as hydrolytic and antagonistic capabilities, which induce beneficial biological responses in hosts, preventing intestinal diseases and microbial infections in farmed fish (Sudhakaran et al. 2022; Rad et al. 2022). Moreover, recent studies have highlighted the influence of modifying culture conditions, including media composition, temperature, and incubation time, on the bioactivity of postbiotics (Garnier et al. 2019; Moradi et al. 2021; Domínguez-Maqueda et al. 2024a). Therefore, optimizing the production of postbiotics offers significant potential for their application in various biotechnological fields, including the aquaculture and aquafeed industry. In this context, microalgae have emerged as a highly promising substrate to be included in bacterial growth media, providing a range of elements to bacterial metabolism that can enhance or maximize the production of ECPs with diverse activities (Ma and Hu 2023; Ricós-Muñoz et al. 2023).

In a previous study conducted by our research group, Medina et al. (2020) selected probiotics based on their ability to generate cross-reactive antibody responses to fish pathogens. Among the evaluated probiotics, *Vibrio proteolyticus* DCF12.2, isolated from healthy wedge sole (*Dicologlossa cuneata*), exhibited remarkable attributes, including its capacity to induce antibody production in fish, inhibit pathogen growth, exhibit non-virulent characteristics toward fish, and maintain viability under storage conditions. Additionally, *V. proteolyticus* DCF12.2 demonstrated diverse hydrolytic activities, such as lecithinase, gelatinase, caseinase, amylase, and lipase, which could potentially contribute to enhance nutrient utilization in fish (Medina et al. 2020). Furthermore, this strain exhibited resilience to passage through the fish gut (Medina et al. 2023). In addition to its in vitro activities, *V. proteolyticus* DCF12.2 induced the expression of immune response-related genes in *Solea senegalensis* and augmented antibody production with cross-reactivity against *Photobacterium damselae* subsp. *piscicida* and *Vibrio harveyi* cells (Medina et al. 2023). Moreover, the strain demonstrated protective effects against challenges posed by *P. damselae* subsp. *piscicida* and *V. harveyi*, highlighting its potential as a preventive measure against diseases caused by these pathogens. However, while the probiotic potential of *V. proteolyticus* DCF12.2 is well recognized (García-Márquez et al. 2023a; García-Marquez et al. 2023b), its postbiotic potential remains to be fully understood. Furthermore, despite some authors have reported the pathogenicity of *V. proteolyticus* strains (Verschuere et al. 2000a; Ray et al. 2016), other strains of *Vibrio proteolyticus* have also been used as probiotics with promising results (De Schrijver and Ollevier 2000; Sugita et al. 2024).

The aim of this study was to investigate how different culture conditions affect the extracellular products (ECPs) secreted by *V. proteolyticus* DCF12.2. The ECPs were evaluated for their hydrolytic, antibacterial, and cytotoxic activities. Furthermore, we analyzed the impact of ECPs on the biofilm formation of several fish pathogens, assessed their DNase and short-chain fatty acid profile, and examined their effect on the relative in vitro transcription of the *aip56* gene, which encodes an important virulence factor of *P. damselae* subsp. *piscicida* (Phdp) (Abushattal et al. 2020). Additionally, a non-targeted metabolomic analysis was conducted on the best ECP condition to further elucidate its metabolic profile.

## Material and Methods

### Bacterial Strain

*Vibrio proteolyticus* DCF12.2 (Medina et al. 2020) was cultured on tryptic soy agar (Oxoid Ltd., Basingstoke, UK) supplemented with NaCl to a final concentration of 2% (TSAs) at 23 °C for 24 h. Then, one to two colonies were cultured on 50 mL of tryptic soy broth (Oxoid Ltd., Basingstoke, UK) supplemented with NaCl to a final concentration of 2% NaCl (TSBs) at 23 °C for 12 h (10^9^ UFC mL^−1^, start of the stationary phase) on shaking at 80 rpm. In addition, the following pathogenic bacterial strains were used: *Aeromonas hydrophila* (Borrego et al. 1991), *Vibrio harveyi* 16/00 (Zorrilla et al. 2003), *Vibrio anguillarum* (CECT 522), *Photobacterium damselae* subsp. *damselae* (CECT 626), and *P. damselae* subsp. *piscicida* (Díaz-Rosales et al. 2003; Arijo et al. 2005). These strains were cultured on TSAs plates at 23 °C for 24 h. Furthermore, *Tenacibaculum maritimum* (CECT 4296), *T. soleae* (CECT 7292), and *T. gallaicum* (CECT 7122) were cultured on *Flexibacter maritimus* medium (FMM) (Condalab, Madrid, Spain) plates supplemented with 1.5% agar at 28 °C for 48 h. All bacterial strains used in this study are listed in Table 1.

**Table 1:** List of bacterial strains used in this study

Bacterial strain	Origin	Use in the study	Reference
*Vibrio proteolyticus* DCF12.2	Healthy wedge sole	Postbiotic production	Medina et al. 2020
*Aeromonas hydrophila*	Disease fish	Antibacterial activity Biofilm inhibition	Borrego et al. 1991
*Vibrio harveyi* 16/00	Diseased Senegalese sole	Antibacterial activity	Zorrilla et al. 2003
*Vibrio anguillarum* CECT 522	Ulcerous lesion in cod (*Gadus morhua*)	Antibacterial activity Biofilm inhibition	-
*Photobacterium damselae* subsp*. damselae* CECT 626	Skin ulcer in damsel fish (*Chromis punctipinnis*)	Antibacterial activity *aip56* gene inhibition	-
*Photobacterium damselae* subsp*. piscicida* Lg41/01	Diseased cultured Senegalese sole	Antibacterial activity	Díaz-Rosales et al. 2003; Arijo et al. 2005
*Tenacibaculum maritimum* CECT 4276	Kidney of diseased black seabream (*Acanthopagrus schlegeli*)	Antibacterial activity Biofilm inhibition	-
*Tenacibaculum soleae* CECT 7292	Diseased sole (*Solea senegalensis*)	Antibacterial activity	-
*Tenacibaculum gallaicum* CECT 7122	Seawater from a holding tank for turbot (*Psetta maxima*)	Antibacterial activity	-

*CECT* Spanish Collection of Type Cultures

### Culture Conditions and Extracellular Product Extraction

Extracellular products (ECPs) from a solid medium were obtained by the cellophane plate technique described by Liu (1957). In brief, 1 mL of the *V. proteolyticus* DCF12.2 culture described above was spread over sterile cellophane sheets placed on TSAs plates (T media). Another 1 mL was spread on sterile cellophane sheets placed on plates containing (i) an experimental aquafeed (160 g L^−1^) and agar (1.5%) (F media), (ii) partial replacement of aquafeed by 25% of a blend of microalgae (*Chlorella fusca*, *Tisochrysis lutea*, *Microchloropsis gaditana*, and *Arthrospira platensis*, 1:1:1:1) (160 g L^−1^) and agar (1.5%) (medium FM), and (iii) total blend of microalgae (*C. fusca*, *T. lutea*, *M. gaditana*, and *A. platensis*, 1:1:1:1) (50 g L^−1^) and agar (1.5%) (medium M). The experimental aquafeed formulations (F and FM media) were elaborated by the Experimental Diets Service (CEIMAR, University of Almeria, Spain) (media composition are shown in Supplementary Table 1). To determine the potential background from the media, cellophane sheets were placed on all media without inoculating the bacterial strain, serving as an internal control. Incubation of all plates was carried out at 15 °C for 48 h and 23 °C for 24 h, as these were the optimum growth temperatures for ECP production, as determined in previous investigations (Domínguez-Maqueda et al. 2024a, 2024b). The different conditions assayed are summarized in Table 2. Each experimental condition was conducted in triplicate to ensure reproducibility. For each replicate, ten individual plates were bulked together, and this process was repeated three times to generate three independent harvests.

**Table 2:** Different culture conditions of *V. proteolyticus* for ECP extraction and nomenclature

Growth media	Temperature and time of incubation	
	**23 °C, 24 h**	**15 °C, 48 h**
Saline tryptic soy agar (T)	T2324	T1548
Aquafeed + agar (F)	F2324	F1548
Aquafeed + 25% microalgae blend + agar (FM)	FM2324	FM1548
Total blend of microalgae + agar (M)	M2324	M1548

After incubation, bacterial cells from the various growth conditions and internal controls were collected in 2 mL sterile phosphate-buffered saline (PBS, pH 7.2) and centrifuged at 10,000 × g for 20 min at 4 °C. The supernatants were then passed through 0.45- and 0.2-µm pore-size membrane filters (Merck Millipore, USA) to obtain the ECPs without cells. The protein concentration was determined using the Qubit Protein assay kits and the Qubit 2.0 fluorometer (Thermo Fisher Scientific, USA). Aliquots of the ECP samples were cultured on TSAs plates and incubated for 24–48 h at 23 °C to confirm the absence of growth. The ECPs were stored at –80 °C until further use.

### Hydrolytic Activity

The protease, collagenase, lipase, and amylase hydrolytic activities were assessed following the methodology described by Chabrillón et al. (2005). These activities were tested on agar plates containing 2% w/v skim milk (Pirinea, Spain), 1% w/v gelatin (Oxoid, UK), 1% w/v Tween-80 (Panreac, USA), and 4% w/v starch (Labkem, USA), respectively. Additionally, phytase, tannase, and cellulase activities were evaluated according to Kumar et al. (2010) on agar plates (1.5% agar) containing 1% w/v sodium phytate (P-8810, Sigma), 2% w/v tannic acid (P-403040, Sigma), and 1% w/v carboxymethyl cellulose (CMC) (C-5678, Sigma), respectively. Wells of 6-mm diameter and 5-mm depth were cut into the agar, and 50 µL of twofold serial dilutions of each ECP sample and internal controls were introduced into the wells. The presence of a clear zone around the wells indicated enzymatic activity. Lipase activity on Tween 80 was confirmed by the appearance of opaque halos of calcium oleate around the wells. Amylase and cellulase activities were confirmed by flooding the plates with Lugol solution and 0.1% w/v Congo red solution, respectively. PBS (50 µL) was used as a negative control. The absence of a clear zone was considered as the absence of enzymatic activity. The lowest concentration of ECP producing a clear/opaque halo was recorded as the minimum effective concentration for each activity. Each ECP condition was tested in triplicate, and experiments were repeated twice.

### Antibacterial Activity

The antibacterial activity of the ECPs was determined using an agar well diffusion assay as described by García-Márquez et al. (2021). The following bacterial strains were used: *Aeromonas hydrophila*, *Vibrio harveyi* 16/00, *Vibrio anguillarum*, *Photobacterium damselae* subsp. *damselae*, and *P. damselae* subsp. *piscicida*. These strains were cultured on TSAs plates at 23 °C for 24 h. Additionally, *Tenacibaculum maritimum*, *T. soleae*, and *T. gallaicum* were cultured on *Flexibacter maritimus* medium (FMM) (Condalab, Madrid, Spain) plates supplemented with 1.5% agar at 28 °C for 48 h. Standardized bacterial suspensions, adjusted to an optical density at 600 nm (OD_600nm_) = 0.1 (∼10^8^ colony-forming units (CFU) mL^−1^), were spread evenly on the surface of TSAs or FMM plates using sterile swabs. To assess the antibacterial activity of the ECPs, 50 µL of each dilution from a series of 1:2 serial dilutions of each ECP sample was introduced into 6-mm-diameter and 5-mm-depth wells on each plate. The starting concentrations for each condition were as follows: T2324: 3150 µg protein mL⁻^1^; F2324: 2760 µg protein mL⁻^1^; FM2324: 3170 µg protein mL⁻^1^; M2324: 2790 µg protein mL⁻^1^; T1548: 2660 µg protein mL⁻^1^; F1548: 2590 µg protein mL⁻^1^; FM1548: 3240 µg protein mL⁻^1^; M1548: 2480 µg protein mL⁻^1^. The dilutions tested ranged from undiluted to 1/2, 1/4, 1/8, and so on until no activity was observed. Internal controls were also included. From these initial concentrations, 50 µL of each sample was introduced into the wells, and the concentration was halved with each subsequent dilution. PBS (50 µL) was used as a negative control. The plates were incubated for 24–48 h at either 23 °C or 28 °C, depending on the optimal growth conditions for each pathogen. The presence of an inhibition halo around each well indicated antibacterial activity. The minimum inhibitory concentration (MIC) was defined as the lowest concentration of ECP that inhibited bacterial growth. Each ECP condition was tested in triplicate, and experiments were repeated twice.

### Cytotoxic Activity

The cytotoxic activity of the ECPs was assessed on fibroblast cells of gilthead seabream (SAF-1, ECACC n°00122301), European seabass brain (DLB-1, CVCL_HG31), *Fundulus heteroclitus* brain (FuB-1, CVCL_YJ47), and hepatocellular carcinoma of *Poeciliopsis lucida* (PLHC-1, ATCC® CRL2406™) cell lines following the methodology fully detailed by Domínguez-Maqueda et al. (2024a). A cytotoxicity assay was conducted in five replicates for each cell type and ECP concentration. In brief, cells at 80% confluence were trypsinized and seeded into 96-well plates at a density of 50,000 cells per well, then incubated for 24 h. The culture medium was replaced with ECPs at concentrations of 0.75, 1, and 1.5 mg mL^−1^ of proteins, while control samples received only culture medium. After a 24-h incubation, cell viability was assessed using the MTT assay (Stevens et al. 1991), which measures the reduction of MTT to formazan by mitochondrial succinate dehydrogenase. The MTT solution (1 mg mL^−1^) was added and incubated for 4 h, and formazan crystals were dissolved in DMSO. Absorbance was read at 570 nm and 690 nm in a microplate reader.

### Biofilm Inhibition Assay

The inhibition of biofilm formation was assessed using the crystal violet (CV) staining method following the methodology described by Domínguez-Maqueda et al. (2024a). *T. maritimum*, *A. hydrophila*, and *V. anguillarum* were selected based on the important role of biofilm formation in their virulence (Croxatto et al. 2007; Rasmussen-Ivey et al. 2016; Mabrok et al. 2023). One colony of each strain was placed in their respective liquid media (FMM for *T. maritimum* and TSBs for *V. anguillarum* and *A*. *hydrophila*) and adjusted to OD_595nm_ ~ 0.1 and transferred into 96-well plates, followed by the addition of either the pathogenic bacterial suspensions alone or with ECP. The biofilm development was assessed after incubation for specific strains. After incubation, biofilm layers were fixed, stained with CV solution, and excess staining was removed and washed with PBS. The OD_595nm_ of the CV, proportional to the number of adherent bacteria, was quantified using a plate reader (Multiskan FC, Thermo Fisher). Growth performance was simultaneously evaluated. Each result was subtracted from the values of control cells, and experiments were conducted in triplicate, with five wells per strain in each assay.

### DNase Activity

DNase activity in the ECPs was determined using DNase test agar plates (Oxoid, UK). In all cases, 50 µL of ECP samples (0.5 µg protein µL^−1^) and the internal controls were inoculated into 6-mm-diameter and 5-mm-depth wells made in the plates and incubated at 23 °C for 24–48 h. The plates were observed for the presence of a clear zone around the wells after flooding the plates with HCl 1 M. As negative control, 50 µL PBS was used. The absence of a clear zone was interpreted as the absence of activity. The lowest ECP concentration with a clear zone around the well was designed as the minimum concentration of DNase activity. Each ECP condition was tested in triplicate, and each experiment was repeated twice.

### Short-Chain Fatty Acid Determination

Short-chain fatty acids (SCFAs) were analyzed as previously described (Barber et al. 2024). The SFCAs for the calibration curve (acetate, propionate, butyrate, iso-butyrate, valerate, and iso-valerate) were obtained from Sigma-Aldrich (St. Louis, MO, USA). A VF-WAXms (30 m × 0.25 mm ID and film thickness of 0.25 µm, Agilent Technologies, Santa Clara, CA, USA) phase capillary column was used with helium as a carrier gas at a constant rate of 1 mL min^−1^. The injector and MS source temperatures were maintained at 200 and 250 °C, respectively. The column temperature was initially set at 90 °C, then increased to 150 °C at 15 °C min^−1^, 170 °C at 5 °C min^−1^, and 250 °C at 20 °C min^−1^ and kept at this temperature for 2 min (total time 14 min). The solvent delay was 3.5 min. The detector operated in electron impact ionization mode (electron energy 70 eV), scanning the 30–250 m/z range. The ion source, quadrupole, and interface temperatures were 230, 150, and 280 °C, respectively. The samples were analyzed in split mode (split ratio: 1/10). The MS operated in the electron impact mode with an ionization energy of 70 eV. The mass scan range was from 50 to 800 Da at 2.05 scan s^−1^.

Data were processed using the Mass Hunter Qualitative Analysis software (version B.10.00, Service Pack 1, Agilent Technologies). To find compounds, the algorithm “compound discovery by deconvolution” was carried out selecting peaks of both GC and MS spectra with an absolute height greater than or equal to 5000 counts. Peak tentative identification was carried out by comparing mass spectra with those of the mass library (Wiley11N17 main/NIST20 mass spectral library; Wiley, Chichester, UK) with a score greater than 80% and comparing the calculated retention indices with those published in the literature.

### *aip56 *Gene Inhibition Assay

#### Minimum Inhibitory Concentration (MIC) of *V. proteolyticus*-ECPs Against Phdp

The minimum inhibitory concentration (MIC) of selected *V. proteolyticus* ECPs, F1548-ECP and FM2324-ECP, against Phdp was determined to assess their potential to inhibit Phdp bacterial growth. Phdp was cultured on TSAs plates at 23 °C for 48 h, and bacterial cells were then collected and suspended in 10 mL TSBs tubes to achieve OD_595nm_ ~ 0.5 (~ 10^9^ UFC mL^−1^). Subsequently, 20 µL of bacterial suspensions was placed into flat-bottom polystyrene 96-well plates (#D51588, Sarstedt, Nümbrecht, Germany), filled up to 200 µL with TSBs, and used as a positive control (Phdp Control +). Concurrently, to determine the MIC of F1548-ECP and FM2324-ECP, 20 µL of Phdp bacterial suspensions was pipetted into microplate wells, and the final volume adjusted to 200 µL by adding 90 µL of double-concentrated TSBs and 90 µL of tenfold dilutions of each ECP (initial protein concentration adjusted to 30 µg mL^−1^) (Phdp + F1548-ECP and Phdp + FM2324-ECP). *V. proteolyticus*-ECPs were added at the beginning of incubation (0 h), and growth was assessed after 48-h incubation by measuring absorbance values (OD_595nm_) using a plate reader (Multiskan FC, Thermo Fisher). The same protocol was followed to determine the inhibitory concentrations of the internal controls (IC) of each *V. proteolyticus*-ECP condition (Phdp + F1548-IC and Phdp + FM2324-IC). Each value was compared with the corresponding control cell values, containing only the culture medium. Three independent experiments were conducted, with five technical replicates (*n* = 5 wells) per condition in each assay.

#### Production of ECPs from Phdp (Phdp-ECPs) Grown in the Presence of *V. proteolyticus*-ECPs

**Fig. 1:**
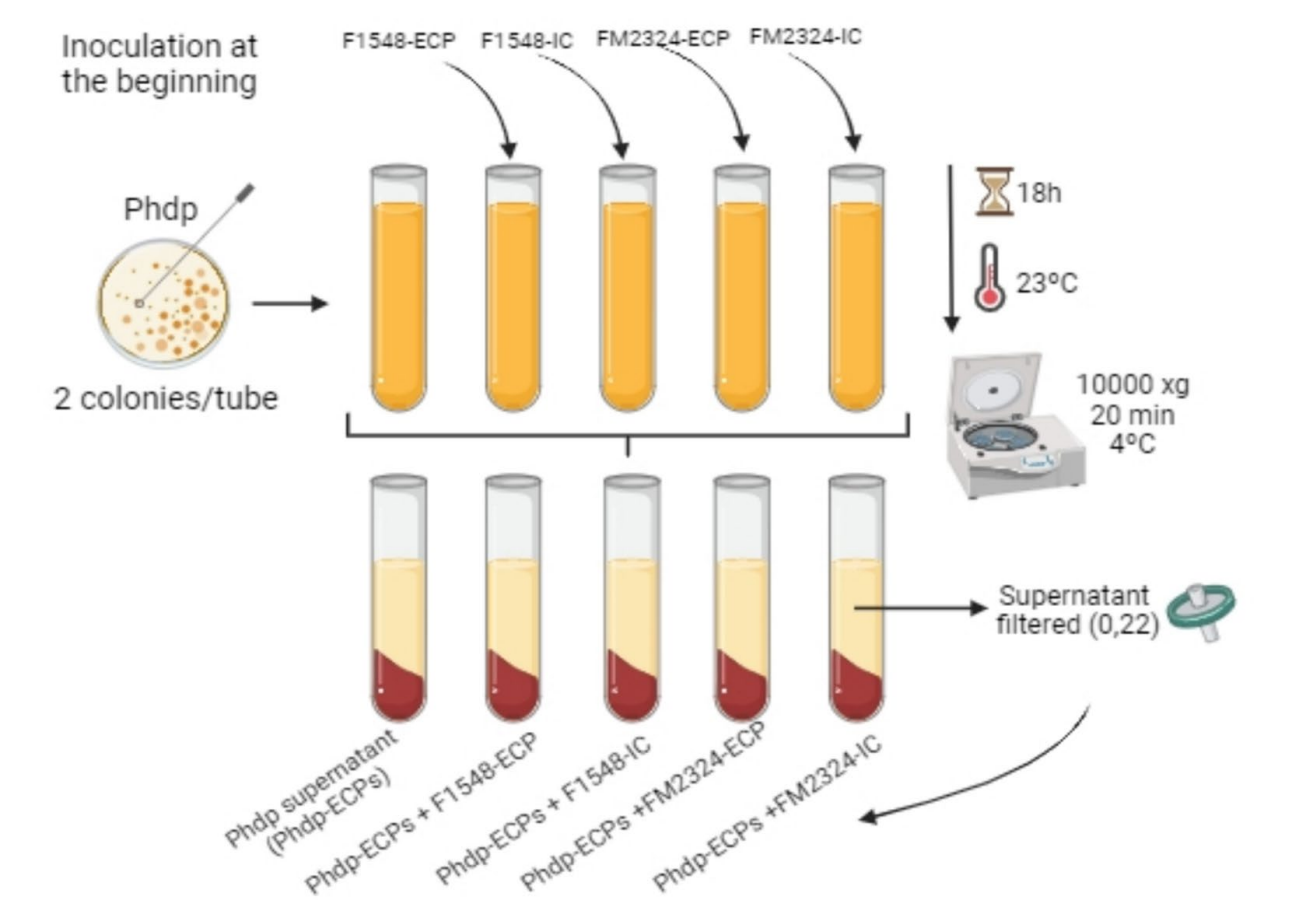
Protocol of extraction of Phdp supernatant alone (Phdp-ECPs) and after adding *V. proteolyticus* DCF12.2 ECPs (F1548-ECP and FM2324-ECP) and controls (F1548-IC and FM2324-IC)

As previously described, Phdp Lg 41/01 was cultured on TSAs plates at 23 °C for 48 h. Following incubation, one or two Phdp colonies were inoculated into 10-mL tubes of TSBs. *V. proteolyticus*-ECP concentration that did not inhibit Phdp growth was selected to obtain Phdp cultured in media containing *V. proteolyticus* ECPs. These selected ECPs were added at the beginning (0 h) (Phdp-ECPs + F1548-ECP and Phdp-ECPs + FM2324-ECP), and incubation was carried out at 23 °C, for 18 h under agitation (120 rpm). Concurrently, 10-mL tubes of Phdp cultures were supplemented with *V. proteolyticus*-ICs and incubated under identical conditions (Phdp-ECPs + F1548-IC and Phdp-ECPs + FM2324-IC). A Phdp culture without *V. proteolyticus*-ECPs or *V. proteolyticus*-ICs was maintained as a positive control (Phdp-ECPs condition) (Fig. 1).

Subsequently, bacterial cells from the different culture conditions were centrifuged (10,000 × g, 20 min, 4 °C), and the resulting supernatants were filtered through 0.45- and 0.2-µm pore-size membrane filters (Merck Millipore, USA). This process aimed to obtain the Phdp supernatant without cells (Phdp-ECPs), with the addition of *V. proteolyticus*-ECPs (Phdp-ECPs + F1548-ECP and Phdp-ECPs + FM2324-ECP) or internal controls (Phdp-ECPs + F1548-IC and Phdp-ECPs + FM2324-IC). The extraction of Phdp-ECPs was necessary to obtain the apoptosis-inducing protein *AIP56*, as it is an extracellular secreted AB-type toxin (Freitas et al. 2022). The protein concentration of the supernatant was determined using Qubit Protein assay kits and the Qubit 2.0 (Thermo Fisher Scientific, USA). To ensure the absence of growth, aliquots of the different ECP samples were cultured on TSAs plates and incubated at 23 °C for 24–48 h. All ECPs were subsequently stored at − 80 °C until further use.

#### Effects of *V. proteolyticus*-ECPs on the Relative Phdp *In Vitro* aip56 Gene Expression

Phdp was cultured on TSAs plates at 23 °C for 48 h, followed by inoculation of one or two Phdp colonies into 10-mL tubes of TSBs. To evaluate the impact of *V. proteolyticus*-ECPs (F1548-ECP and FM2324-ECP) and their internal controls (F1548-IC and FM2324-IC) on the relative in vitro expression of the Phdp *aip56* gene, selected dilutions of F1548-ECP and FM2324-ECP that did not inhibit bacterial growth were added to TSBs tubes at the beginning of incubation (0 h) and incubated at 23 °C for 18 h under shaking (120 rpm). Simultaneously, 10-mL tubes of Phdp cultures were added with the internal controls (F1548-IC and FM2324-IC) and subjected to identical incubation conditions. A Phdp culture without *V. proteolyticus*-ECPs or *V. proteolyticus*-ICs was maintained as a positive control. After 18 h of incubation, cells were harvested by centrifugation at 5000 × g for 10 min at 4 °C. Three independent experiments were conducted, each with five technical replicates (*n* = 5 wells).

Subsequently, RNA extraction from the bacterial cells was performed using the RNA Purification Kit (#K0731 Thermo Scientific™) following the manufacturer’s instructions. RNA quality was assessed using the 2 × RNA Loading Dye kit (#R0641 Thermo Scientific™), with 2 × loading buffer added to 2 µL of previously extracted RNA, followed by heat shock at 95 °C for 5 min, and analysis on agarose gels (1% w/v). Quantification of extracted RNA was conducted using the Qubit 2.0 High Sensitivity quantification kit (Thermo Scientific, Madrid, Spain), with RNA stored at − 80 °C until further use. Subsequently, cDNA was synthesized from 100 ng RNA of each sample using the Maxima First Strand cDNA Synthesis Kit for RT-qPCR with dsDNase (#K1671 Thermo Scientific), and stored at − 20 °C.

The relative transcription of the *aip56* gene was determined using quantitative reverse transcription PCR (qRT-PCR). Each reaction mixture contained 2 µL of cDNA, 50 U of Taq Accustart II Trough Mix (Biomerieux, Marcy-l’Étoile, France), 20 pmol of *aip56*_R primer (5′-CGGCAGTGAATTAGGCTTTCT-3′), and 20 pmol of *aip56*_F primer (5′-CCGCCTCCGTTGAAATCATCC-3′) in a final volume of 20 µL. The primers were obtained from Nuñez-Díaz et al. (2018). The PCR program included an initial denaturation at 95 °C for 60 s, followed by 40 cycles of 95 °C for 30 s, 55 °C for 40 s, and 72 °C for 60 s. Amplification was followed by a standard melting curve from 65 to 95 °C, in increments of 0.5 °C for 5 s at each step. Samples were run in parallel with the *16S* rRNA reference gene. RT-qPCR reactions were performed on a CFX96 Touch Real-Time PCR Detection System (Bio-Rad Laboratories, Hercules, CA, USA). Relative fold change values were calculated using the comparative Cq method (2^−ΔΔCt^) (Livak and Schmittgen 2001), normalizing gene expression from different growth conditions to the reference gene and expressing values relative to Phdp cells grown in TSBs.

### Untargeted Metabolomics

The untargeted metabolomics analysis was conducted using ultra-high performance liquid chromatography with quadrupole time-of-flight mass spectrometry (UPLC-Q-TOF–MS) in both positive and negative ionization modes, following the methodology fully described by Barber et al. (2021) and Barber et al. (2024). The UPLC-Q-TOF system included an Agilent 1290 Infinity LC system coupled to a 6550 I-Funnel QTOF with a dual electrospray ionization interface for continuous calibration of m/z ratios. Samples were injected into a Poroshell 120 EC-C18 column and separated using a gradient of acidified water and ACN. Data acquisition was managed by Mass Hunter Workstation software with settings optimized for high selectivity, sensitivity, resolution, and mass accuracy. The system was calibrated both at the beginning of the analysis and continuously during the analysis. MS/MS conditions included a collision energy of 20 eV and an acquisition time of 100 ms/spectrum. All samples were injected in a randomized order to avoid bias, with quality control and blank runs included to ensure accuracy and avoid carry-over effects.

The raw data files were acquired in profile file mode and exported to MZmine software (Version 2.53, Copyright (c) 2005–2015 MZmine Development Team) to create the data matrix. Raw data were pre-processed by a batch set of parameters including the mass detection, chromatogram builder, and deconvolution and alignment algorithm. The data matrix was exported to Mass Profiler Professional (MPP, Agilent Technologies, Waldbronn, Germany) and Metaboanalyst 5.0 online platform (https://www.metaboanalyst.ca/) for parallel data management. The data matrix was compared to the Human Microbial Metabolome Database (MiMeDB) (Wishart et al. 2023) for further validation, with selections based on the score and their biological significance within the study context. All assays were performed with three replicates per sample. ECP conditions were compared using *t*-tests and Wilcoxon rank-sum tests on the MetaboAnalyst 5.0 platform, with a *p*-value threshold of 0.05. Subsequently, log_2_ (Fold Change, FC) and − log10 (*p*-value) were established and represented on a volcano plot after FDR correction. The specific and common metabolites of each ECP condition were depicted in a Venn diagram. The raw dataset can be downloaded from the Mass Spectrometry Interactive Virtual Environment (MassIVE) online repository at https://massive.ucsd.edu/ProteoSAFe/static/massive.jsp using the dataset identification MSV000095403.

### Statistical Analysis

Statistical analyses were performed using GraphPad Prism 9. The normality and homogeneity of variance of the data were assessed using the Shapiro–Wilk and Levene’s tests, respectively. Differences between groups were evaluated using one-way analysis of variance (ANOVA), followed by Tukey and Games-Howell post hoc tests when the assumptions of normality and homogeneity of variance were met. For non-normally distributed data, the Kruskal–Wallis test was employed, followed by a multiple comparison test. DNase activity and SCFA composition were evaluated using a Student’s *t*-test. Statistical significance was established at *p* ≤ 0.05.

## Results

### Hydrolytic and Antibacterial Activity of ECPs

The hydrolytic and antibacterial activities of the ECPs from *V. proteolyticus* DCF12.2 were evaluated, and the results are summarized in Table 3. Enzymatic activity assays revealed that ECPs obtained under all conditions were capable of hydrolyzing starch, gelatin, and casein. However, only ECPs obtained under four conditions (T1548, M1548, FM2324, and M2324) exhibited lipase activity. None of the ECPs demonstrated the ability to hydrolyze cellulose, tannins, or phytate. Regarding the antibacterial activity of the ECPs, *P. damselae* subsp. *piscicida* was inhibited by ECPs recovered from F and FM media, whereas *P. damselae* subsp. *damselae* was inhibited by ECPs recovered from FM media, regardless of temperature and incubation time. No inhibitory effects were observed against the other tested pathogens (Table 3). The internal controls did not exhibit any hydrolytic enzyme activity or antibacterial effects.

**Table 3:** Hydrolytic and antibacterial activities of ECP samples extracted from different conditions. The results are expressed as the mean ± SD of the minimum concentration in the ECPs for each activity (µg protein mL^−1^). –, no activity

	**T2324**	**F2324**	**FM2324**	**M2324**	**T1548**	**F1548**	**FM1548**	**M1548**
Hydrolytic activity								
**Amylase**	3,100.0 ± 50.0^a^	691.0 ± 17.0^c^	49.5 ± 0.8^ g^	87.3 ± 1.9^f^	1,300.0 ± 20.0^b^	635.0 ± 11.3^d^	97.5 ± 2.5^e^	37.9 ± 1.0^ h^
**Collagenase**	96.9 ± 1.6^a^	43.2 ± 1.1^c^	24.8 ± 0.4^e^	43.6 ± 0.9^c^	81.3 ± 1.3^b^	79.4 ± 1.4^b^	97.5 ± 2.5^a^	37.9 ± 1.0^d^
**Caseinase**	96.9 ± 1.6^a^	43.2 ± 1.1^f^	49.5 ± 0.8^e^	87.3 ± 1.9^b^	81.3 ± 1.3^c^	79.4 ± 1.4^c^	97.5 ± 2.5^a^	75.8 ± 2.0^d^
**Lipase**	–	–	792 ± 12.0^b^	698.0 ± 15.0^c^	2,600.0 ± 40.0^a^	–	–	606.0 ± 16.3^d^
**Phytase**	–	–	–	–	–	–	–	–
**Cellulase**	–	–	–	–	–	–	–	–
**Tannase**	–	–	–	–	–	–	–	–
Antibacterial activity								
***A. hydrophila***	–	–	–	–	–	–	–	–
***V. harveyi***	–	–	–	–	–	–	–	–
***V. anguillarum***	–	–	–	–	–	–	–	–
***P. damselae subsp. damselae***	–	–	792.0 ± 12.0^b^	–	–	–	3,120.0 ± 80.0^a^	–
***P. damselae subsp. piscicida***	–	1,382.0 ± 34.0^c^	792.0 ± 12.0^d^	–	–	2,540.0 ± 45.0^b^	3,120.0 ± 80.0^a^	–
***T. maritimum***	–	–	–	–	–	–	–	–
***T. soleae***	–	–	–	–	–	–	–	–
***T. gallaicum***	–	–	–	–	–	–	–	–

### Cytotoxicity of ECPs

**Fig. 2:**
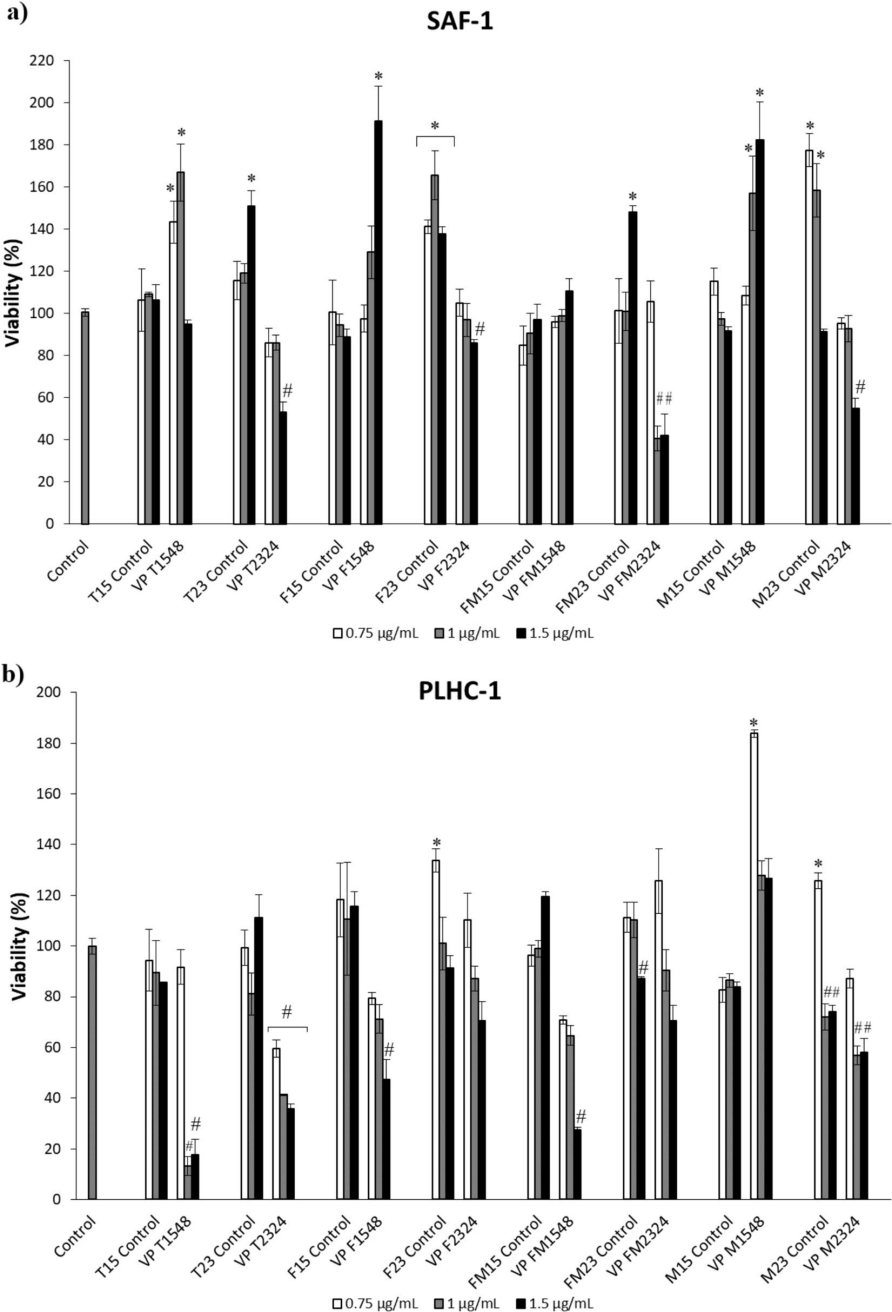

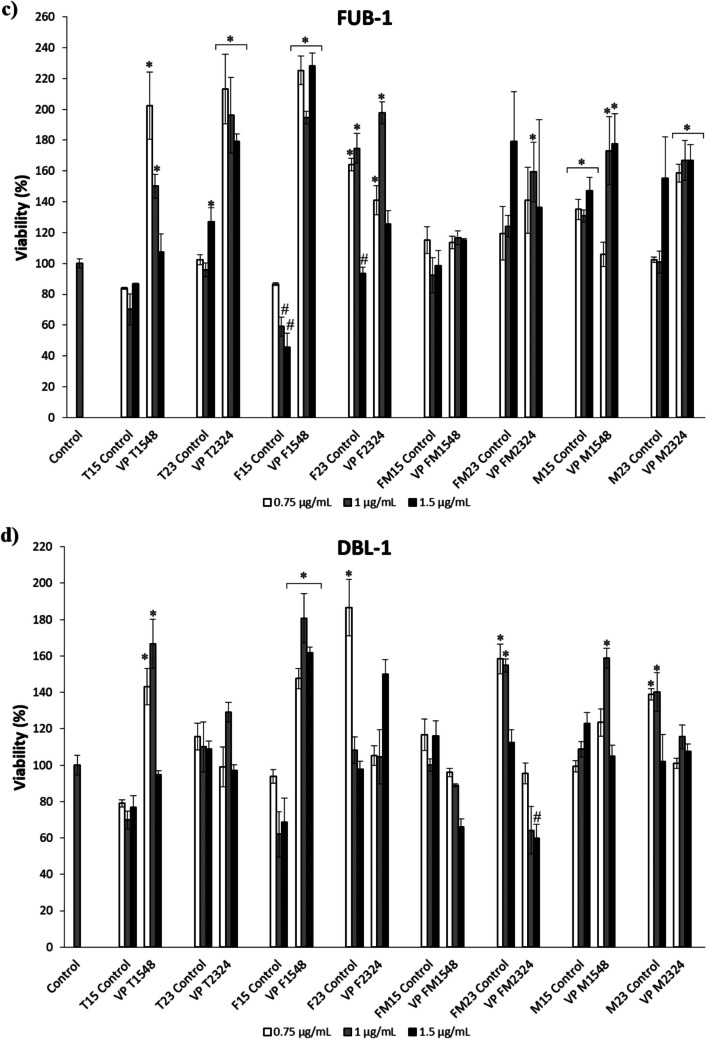
Cytotoxic effect produced by ECPs of *V. proteolyticus* DCF12.2 on various cell lines. **a** Fibroblast cell line (SAF-1) (*Sparus aurata*), **b** fish hepatoma cell line (PLHC-1) (*Poeciliopsis lucida*), **c** brain cell line (FuB-1) from mummichog (*Fundulus heteroclitus*), **d** brain cell line (DLB-1) from European seabass (*Dicentrarchus labrax*). The increase in viability was detected after 24 h of incubation. The concentrations of ECPs tested on all cells were 0.75, 1, and 1.5 µg mL.^−1^. The values reported are the means of three replicates. Hash marks (#) or asterisks (*) indicate reduction and proliferation, respectively, of cell viability (*p* < 0.05)

The cytotoxic activity of ECPs derived from *V. proteolyticus* DCF12.2, as well as their respective internal controls, was evaluated using various cell lines (Fig. 2). For SAF-1 cells, ECPs obtained under conditions T1548, F1548, and M1548 increased cell viability, while all ECP conditions obtained at 23 °C for 24 h (T2324, F2324, FM2324, and M2324) reduced cell viability (Fig. 2a). In general, the ECPs from *V. proteolyticus* were cytotoxic to the PLHC-1 cell line, except for the M1548 condition, which promoted cell viability (Fig. 2b). In contrast, ECPs from *V. proteolyticus* significantly increased the viability of FuB-1 cells (Fig. 2c). Lastly, the ECPs from *V. proteolyticus* did not exhibit cytotoxic effects on the DLB-1 cell line, except for specific conditions such as FM2324 (Fig. 2d). Additionally, some of the concentrations tested for ECPs (T1548, F1548, and M1548) significantly increased cell viability.

Based on the hydrolytic, antibacterial, and cytotoxic activities of the ECPs, we selected F1548 and FM2324 (and their internal controls) for further characterization.

### Biofilm Production

**Fig. 3:**
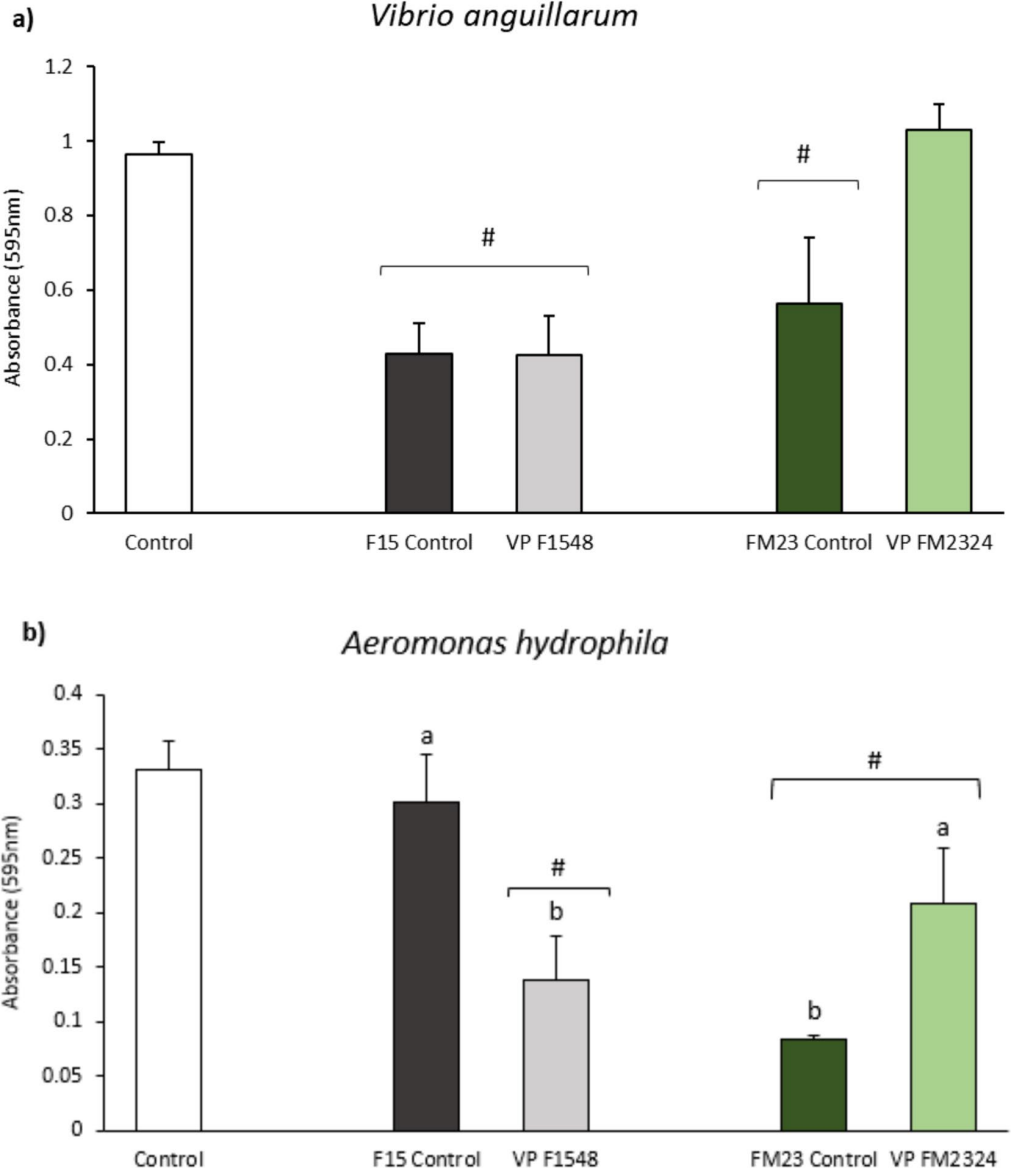

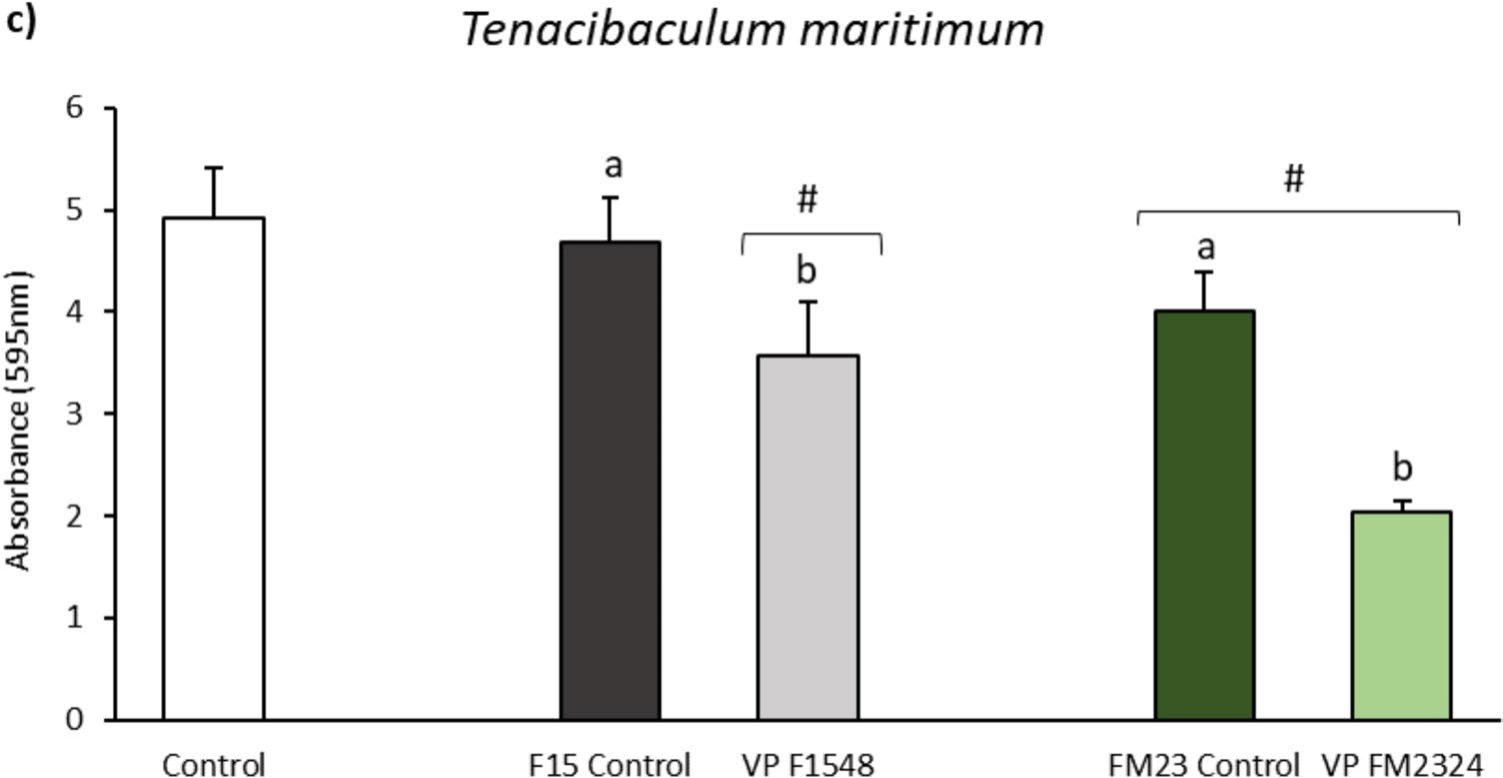
Inhibition of biofilm formation: **a** *V. anguillarum*, **b** *A. hydrophila*, and **c** *T. maritimum* by the different ECP samples. White bars represent the biofilm formation of each bacterium (control group). The results are representative of three independent experiments and are expressed as mean ± SD (*n* = 5). Hash marks (#) and asterisks (*) indicate reduction and proliferation, respectively, of biofilm formation between treatments and bacterial control (*p* < 0.05). Different letters indicate significant differences between treatments and their internal controls (*p* < 0.05)

The effects of different ECPs and their respective internal controls on biofilm formation by various fish pathogens are presented in Fig. 3. The results indicate that ECPs from F media (F1548 and F15 Control) and F23 Control significantly reduced biofilm formation by *V. anguillarum* (Fig. 3a). Conversely, the FM2324 ECP condition significantly increased biofilm formation by *V. anguillarum* compared to its internal control (FM23 Control).

For *A. hydrophila* and *T. maritimum*, the ECPs from FM media (FM2324 and F23 Control) and ECP condition F1548 significantly reduced biofilm formation (Fig. 3b and 3c, respectively). Additionally, while the ECP condition F1548 significantly decreased the biofilm formation of *A. hydrophila* and *T. maritimum* relative to its internal control (F15 Control) (Fig. 3b and 3c, respectively), ECP condition FM2324 increased the biofilm of *A. hydrophila* and decreased the biofilm of *T. maritimum* relative to its internal control (FM23 Control) (Fig. 3b and 3c, respectively).

### DNase Activity and Short-Chain Fatty Acid Composition

DNase activity was observed in ECPs from *V. proteolyticus* DCF12.2 at nearly the same concentrations (103.5 µg protein mL^−1^ for F1548 and 102.5 µg protein mL^−1^ for FM2324) (Table 4). Regarding the short-chain fatty acid composition, both ECPs contained acetic, propionic, and iso-valeric acids (with a statistically higher concentration in FM2324 than in F1548) but did not contain iso-butyric acid (Table 4). Additionally, FM2324 had butyric and valeric acids. Finally, no DNase activity or short-chain fatty acids were detected in the internal controls.

**Table 4:** DNase activity and short-chain fatty acid composition of selected ECPs of *V. proteolyticus* DCF12.2. Data are presented as mean ± SD of three replicates. Asterisks indicate significant differences between ECP conditions (*p* < 0.05). *Nd*, not detected

	**ECP conditions**	
	**F1548**	**FM2324**
DNase (µg protein mL^−1^)	103.5 ± 2.1	102.5 ± 1.9
*Short-chain fatty acids*		
Acetic acid (µM)	4.16 ± 0.2	3.71 ± 0.3
Propionic acid (µM)	0.10 ± 0.0	0.10 ± 0.0
Iso-butyric acid (µM)	Nd	Nd
Butyric acid (µM)	Nd	0.90 ± 0.0
Iso-valeric acid (µM)	1.35 ± 0.3	4.81 ± 0.2*
Valeric acid (µM)	Nd	0.09 ± 0.0

### Effect of ECPs on Phdp aip56 Gene Expression

**Fig. 4:**
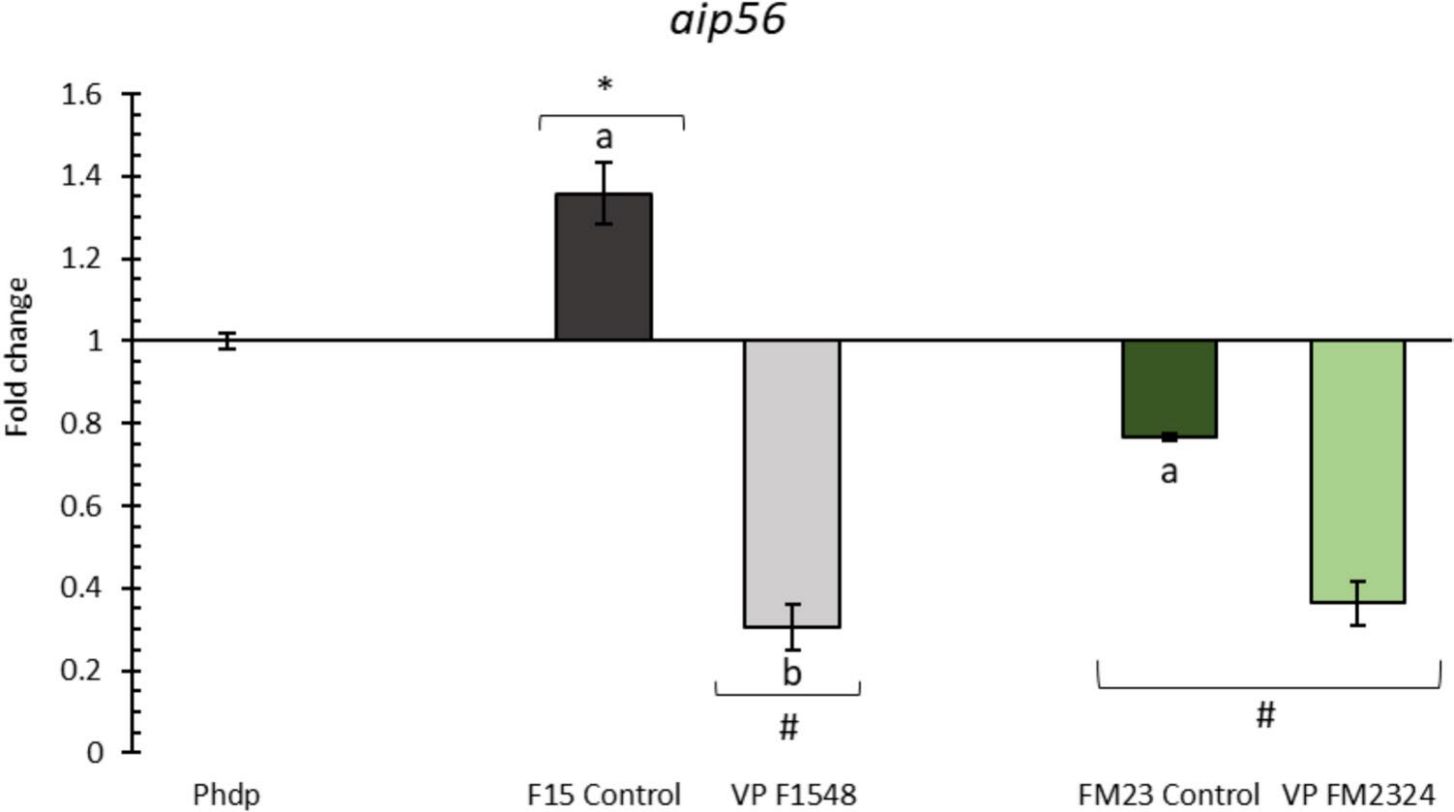
Relative expression of the Phdp *aip56* gene. In all cases, expression values are shown for internal controls and ECP treatments of the selected strains. Letters (a, b) indicate significant differences (one-way ANOVA; *p* < 0.05) between treatments and their internal controls. Asterisks (*) and hash marks (#) indicate upregulation or downregulation, respectively (one-way ANOVA; *p* < 0.05) compared to the control (Phdp). Values represent the mean ± standard error of the mean (SEM) of four technical replicates and two biological replicates

ECPs from *V. proteolyticus* DCF12.2 (F1548 and FM2324) significantly reduced the expression of the *aip56* gene compared to both the control (Phdp) and their respective internal controls (F15 and FM23 Control) (Fig. 4).

Based on the overall activity, we selected F1548 (and their internal controls) for further characterization at the metabolomic level.

### Metabolomic Study of Selected ECPs

**Fig. 5:**
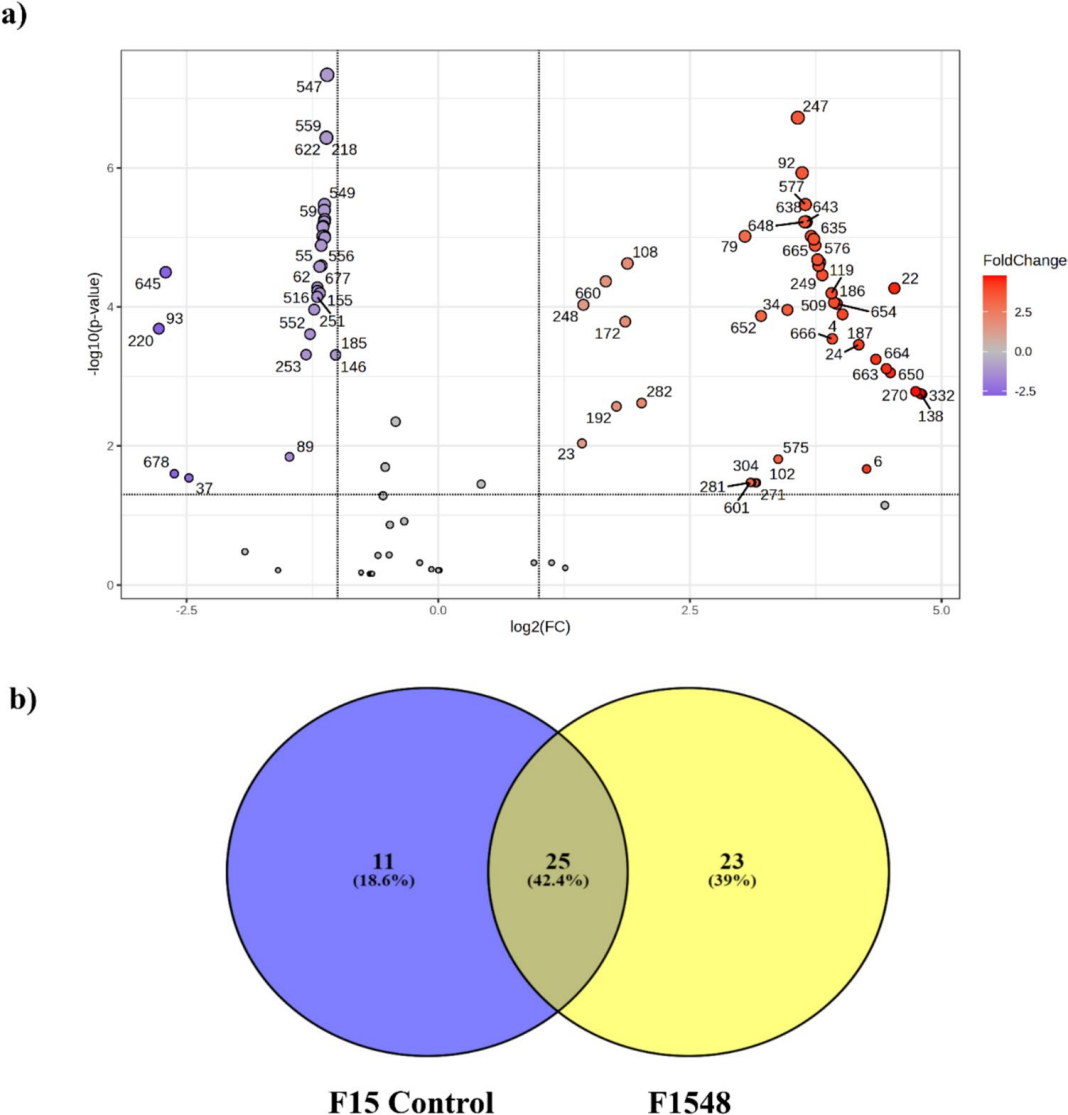
**a** Volcano plots showing the distribution of identified metabolites in ECPs from condition F1548 compared to F15 Control, with the − log_10_ of adjusted *p*-values plotted against log_2_ (fold change). The two vertical dotted black lines represent log_2_ fold changes of − 0.5 and 2, while the horizontal dotted line indicates the significance threshold of − log_10_ (*p* ≤ 0.05). In the plot, red dots represent upregulated metabolites, purple dots represent downregulated metabolites, and grey dots represent non-differentially expressed metabolites. **b** Venn diagram illustrating the distribution of all identified differentially expressed metabolites, showing those unique to each condition (F15 Control (blue) and F1548 (yellow)) and those common to both conditions

The metabolite content of ECPs from *V. proteolyticus* F1548 and the internal control (F15 Control) was assessed. Volcano plots indicated differential expression of metabolites (DEMs) in the F1548 ECP condition (Fig. 5a). Among these DEMs, 34 were downregulated (purple), 47 were upregulated (red/pink), and 37 were not significantly altered compared to the F15 Control (grey). Furthermore, Venn diagram analysis, based on a differential abundance threshold of ≤ 1.3, identified 25 common metabolites, constituting the -metabolome core (Fig. 5b).

The F1548 condition was characterized by 23 unique metabolites, including amino acids and derivatives such as acetyl-L-leucine, citrulline, DL-histidinol, L-2-aminoadipic acid, L-ornithine, L-phenylalanine, L-valine, N-acetyl-L-glutamic acid, N-acetyl-L-histidine, and N-acetyl-L-phenylalanine; sugars and sugar derivatives like D-ribose and 7-methyl-1,4,5-naphthalenetriol 4-[xylosyl-(1- > 6)-glucoside]; nucleotides and nucleosides including uridine and uridine monophosphate (UMP); organic acids such as 2,5-dioxopentanoate, glutaric acid, propionic acid, and uric acid; lipids like cholic acid; and other compounds including (2S,3′S)-alpha-amino-2-carboxy-5-oxo-1-pyrrolidinebutanoic acid, N1-(alpha-D-ribosyl)−5,6-dimethyl-benzimidazole, phenolphthalin, and trans-cinnamic acid.

## Discussion

Members of the genus *Vibrio* are ubiquitous in marine environments. While certain species, including *V. proteolyticus*, have been reported as pathogenic (Verschuere et al. 2000a; Ray et al. 2016; Zhang et al. 2020; Manchanayake et al. 2022), others—*V. proteolyticus* among them—have also been proposed as probiotics (De Schrijver and Ollevier 2000; Verschuere et al. 2000b; Thompson et al. 2010; Sheikh et al. 2022). The probiotic potential of the *V. proteolyticus* strain DCF12.2 used in this study lies in its ability to produce beneficial extracellular enzymes that enhance nutrient availability and inhibit bacterial pathogens, as demonstrated by our findings (Medina et al. 2020, 2023).

The enzymatic activity assays revealed that the ECPs from *V. proteolyticus* DCF12.2 maintained a broad range of hydrolytic activities that were exhibited in the cells (Medina et al. 2020). Specifically, all tested conditions could hydrolyze starch, gelatin, and casein, indicating persistent enzymatic activity in the ECPs. However, lipase activity was only observed in four conditions: T1548, M1548, FM2324, and M2324.

Amylase, caseinase, and collagenase activities were consistently found across all ECP conditions. These enzymes play a crucial role in nutrient digestion and absorption in fish. Amylase breaks down starch into simpler sugars, enhancing carbohydrate availability (Mardani et al. 2018), while proteolytic activities like gelatinase and caseinase can improve feed digestibility and nutrient absorption (Gajanan et al. 2016; Singh and Benjakul 2018). Previous studies have shown that pre-digested protein improves growth performance, nutrient utilization, intestinal microbiota, and immune response in fish (Swanepoel and Goosen 2018; Rimoldi et al. 2020). On the other hand, elevated proteolytic activity in gut microbiota can also increase intestinal permeability and inflammation (Barbara et al. 2021; Gieryńska et al. 2022). Lipase activity, which was only detected in the T1548, M1548, FM2324, and M2324 conditions, plays a significant role in lipid metabolism, aiding in the hydrolysis of triglycerides and the absorption of lipid droplets (Cabodevilla et al. 2024), supporting efficient feed utilization and energy uptake (Bakke et al. 2010). Further in vivo studies are needed to corroborate these benefits and include histological analyses.

Postbiotics could offer potential solutions to the emergence of multidrug-resistant bacteria due to the widespread use of antibiotics. The results demonstrated specific antibacterial effects under certain conditions. ECPs recovered from F and FM media inhibited *P. damselae* subsp. *piscicida*, regardless of temperature and incubation time. Furthermore, *P. damselae* subsp. *damselae* was inhibited only by ECPs from FM media. These findings align with previous research showing that *V. proteolyticus* cells possess inherent antibacterial capabilities against fish pathogens, including *P. damselae* subsp. *piscicida* (Medina et al. 2020). Notably, no antibacterial activity was observed in ECPs from the TSAs medium, indicating that feed (F media) and feed supplemented with microalgae (FM media) influence the production of antibacterial metabolites. This indicates a potential synergistic effect between the nutrients and compounds present in the media (specifically in F and FM media) and the bacteria, possibly promoting the production of bioactive metabolites with antibacterial properties. This hypothesis is further supported by the lack of antibacterial activity in the internal controls, which may not contain these metabolites.

The cytotoxic activity of the ECPs was evaluated using various fish cell lines, revealing distinct effects based on the conditions under which the ECPs were produced. For the SAF-1 cell line, derived from gilthead seabream fibroblasts, ECPs from T1548, F1548, and M1548 increased cell viability, while all ECPs produced at 23 °C for 24 h (T2324, F2324, FM2324, and M2324) reduced viability. This temperature-dependent cytotoxicity aligns with previous findings that growth temperature can influence bacterial cytotoxicity through structural changes in cytotoxic proteins (Nsonzi et al. 2015; Briaud et al. 2021). Similarly, our results indicate lower cytotoxicity on SAF-1 cells when ECPs were recovered at 15 °C compared to 23 °C. This observation was also observed by Domínguez-Maqueda et al. (2024a), who found higher cytotoxicity in ECPs from *Shewanella putrefaciens* Pdp11 at 23 °C than at 15 °C. These findings highlight the importance of evaluating different growth conditions, such as temperature, in modulating the secretion and activity of ECPs.

Interestingly, ECPs exhibited a proliferative effect on fish brain cells (FuB-1, derived from *F. heteroclitus*, and DBL-1, derived from *D. labrax*), suggesting neuroprotective properties or promotion of cell proliferation in brain-derived cell lines. The potential beneficial effects on brain cells could have significant implications for understanding how these ECPs interact with critical biological processes such as reproduction, behavior, growth, feeding, and circadian rhythmicity, in which the brain plays essential integrative and effector roles (Papoutsoglou 2012; Fernö et al. 2020; Miletto Petrazzini et al. 2020). Conversely, most ECP conditions from exhibited cytotoxic effects on the tumor PLHC-1 cell line, indicating a potential specific cytotoxic effect against tumor cells. Further studies are needed to elucidate the molecular mechanisms behind these effects, which could lead to new anticancer agents for use in aquaculture, veterinary, or human medicine.

Based on the hydrolytic, antibacterial, and cytotoxic activities of the ECPs, we selected F1548 and FM2324 (and their internal controls) for further characterization.

Biofilm formation is a critical factor in the pathogenicity of many bacterial species, providing protection against environmental stressors and antibiotics (Rather et al. 2021). The ECPs derived from the F medium, specifically conditions F1548 and F15 Control, as well as F23 Control, significantly reduced biofilm formation by *V. anguillarum*. This reduction is particularly notable for F1548, which effectively diminished biofilm formation across all three tested pathogens (*V. anguillarum*, *Aeromonas hydrophila*, and *Tenacibaculum maritimum*). Conversely, the FM2324 ECP condition significantly increased biofilm formation by *V. anguillarum* compared to its internal control (FM23 Control). However, this same condition (FM2324) significantly reduced biofilm formation for both *A. hydrophila* and *T. maritimum*. This differential response indicates a complex interaction between the ECPs, the specific pathogen, and the conditions under which the ECPs were produced.

The differential effects on biofilm formation may be related to the enzymatic activities present in these preparations. Both F1548 and FM2324 conditions exhibited DNase activity, which was absent in their respective internal controls. DNases are known to degrade extracellular DNA, a key component of the biofilm matrix, thereby disrupting biofilm integrity and promoting its dispersal (Jakubovics et al. 2013; Okshevsky and Meyer 2015). Furthermore, the proteolytic activity of ECPs may also play a role in modulating biofilm formation. Proteolytic enzymes can degrade extracellular polymeric substances (EPS) that provide structural stability to biofilms (Borges et al. 2020; Li et al. 2022). The significant biofilm reduction by F1548, with both DNase and proteolytic activities, highlights the potential of these enzymatic actions in biofilm control. However, the increase in biofilm formation by *V. anguillarum* under FM2324 suggests other factors or interactions may be involved, possibly related to specific FM medium components or differential pathogen responses.

The short-chain fatty acid (SCFA) profile of the ECPs was also assessed due to their potential benefits as feed additives in aquaculture (Hoseinifar et al. 2017). Both ECP samples analyzed, F1548 and FM2324, consistently contained acetic, propionic, and iso-valeric acids but lacked iso-butyric acid. Additionally, the FM2324 sample exhibited the presence of butyric and valeric acids, further diversifying its SCFA profile. Acetic acid, a common SCFA, is well-documented for its antimicrobial properties and role in metabolic processes (Rauf et al. 2022; Hosmer et al. 2024). Its presence in both ECPs suggests a potential baseline level of antimicrobial activity, which could be beneficial in various biotechnological applications, including aquaculture. Iso-valeric acid, another SCFA found in both ECPs, is typically associated with protein fermentation (Cho et al. 2015; Bevilacqua et al. 2022). Its presence aligns with the observed proteolytic activity of *V. proteolyticus* DCF12.2, supporting the notion that these ECPs could enhance the breakdown of proteinaceous materials. This could have practical implications for improving nutrient bioavailability in feed formulations.

Notably, butyric acid, present in FM2324, is recognized for its anti-inflammatory properties and role in maintaining gut health (Zou et al. 2019; Zhu et al. 2021), suggesting additional benefits for fish growth and resilience to infections. Valeric acid, also in FM2324, has been shown to have similar benefits to butyric acid, including antimicrobial and gut health-promoting effects (Kovanda et al. 2019; Gao et al. 2022). The combination of butyric and valeric acids in FM2324 may thus confer enhanced protective effects, making this ECP particularly promising for future applications.

*P. damselae* subsp. *piscicida* is the causative agent of photobacteriosis or pasteurellosis (Romalde 2002), a prevalent disease affecting various marine fish species, such as gilthead seabream (*S. aurata*), European seabass (*D. labrax*), and Senegalese sole (*S. senegalensis*) (Fumanal et al. 2020; Santos et al. 2022; Valsamidis et al. 2023). *P. damselae* subsp. *piscicida*, including the strain studied here, *P. damselae* subsp. *piscicida* Lg41/01 (Phdp), has the capability to invade non-phagocytic cells and evade the immune response (Acosta et al. 2009). Moreover, all virulent strains carry a plasmid containing the *aip56* toxin gene (Abushattal et al. 2020), which encodes the AIP56 exotoxin capable of inducing apoptosis of fish macrophages and neutrophils (Freitas et al. 2022). In the present study, all ECP conditions resulted in downregulation of *aip56* gene transcription, corroborating findings by Domínguez-Maqueda et al. (2024b). In their research, ECPs derived from *Shewanella putrefaciens* Pdp11 cultured on tryptone soy agar, supplemented with a partial replacement of aquafeed by 25% of a blend of microalgae and cyanobacteria (*C. fusca*, *T. galbana*, *M. gaditana*, and *A. platensis*), similarly downregulated *aip56* gene expression in Phdp. Additionally, Medina et al. (2023) demonstrated that Senegalese sole fed with *V. proteolyticus* exhibited higher survival rates compared to a control group when challenged with *P. damselae* subsp. *piscicida*. Collectively, these findings suggest that both the cells and ECPs of *V. proteolyticus* may be effective in mitigating the pathogenicity of *P. damselae* subsp. *piscicida*. Further research is needed to understand the mechanisms behind this gene regulation.

Based on the overall activity, we selected F1548 (and its internal controls) for further characterization at the metabolomic level. Several metabolites identified exclusively in the F1548 condition have known biological activities that could contribute to these effects. Among the unique metabolites, propionic acid has well-documented antimicrobial properties. Propionic acid is known for its broad-spectrum antimicrobial activity, particularly against Gram-positive bacteria (Wang et al. 2014). It disrupts cellular processes by penetrating the bacterial cell wall and lowering intracellular pH, which can inhibit bacterial growth and survival (Ricke 2003). Additionally, the presence of several amino acids and derivatives exhibits promising antimicrobial and antioxidant activity (Kasai et al. 2015, 2021).

Trans-cinnamic acid, another unique metabolite, is a hydrophobic, phenolic compound with antimicrobial, antioxidant, anti-cancer, and anti-inflammatory properties (Edis et al. 2020). It has been shown to disrupt the biofilm formation of *S. aureus*, *S. typhimurium*, and *P. aeruginosa* (Letsididi et al. 2018). Glutamic acid, also found in the F1548 condition, has been found to inhibit and disperse *S. aureus* NCTC 8325 biofilms (Warraich et al. 2020). Thus, the unique metabolite profile of the F1548 condition reflects a complex interplay of compounds that collectively contribute to the observed activities. The presence of diverse metabolites such as organic acids, amino acids, and nucleotides indicates a multifaceted approach to inhibiting bacterial growth and pathogenicity. This holistic effect underscores the potential of using ECPs from *V. proteolyticus* DCF12.2 as an effective strategy for combating bacterial infections and biofilm-related issues in aquaculture and clinical settings.

In conclusion, the ECPs from *V. proteolyticus* DCF12.2 exhibit diverse and beneficial biological activities, including enzymatic digestion, antibacterial properties, biofilm modulation, reduction of virulence gene expression, and variable cytotoxic effects depending on the cell type. These findings highlight the potential of ECPs as valuable additives in aquaculture to enhance fish nutrition, health, and disease resistance. Future research should aim to isolate and characterize the specific bioactive compounds responsible for these effects and elucidate their mechanisms of action to optimize their application in aquaculture and potentially other biotechnological fields.

## Supplementary Information

Below is the link to the electronic supplementary material.

Supplementary file1 (DOCX 17 KB)

## Data Availability

Metabolomic data can be downloaded from the Mass Spectrometry Interactive Virtual Environment (MassIVE) online repository at https://massive.ucsd.edu/ProteoSAFe/static/massive.jsp using the dataset identification MSV000095403.
