# Supplementary material for: Optimizing Extracellular Products from Vibrio proteolyticus for Their Use as Postbiotics in Aquaculture
Source: Mar Biotechnol (NY). 2025 Aug 2;27(4):120. doi: 10.1007/s10126-025-10500-6 (PMC12317916; doi:10.1007/s10126-025-10500-6)
Supplement: Supplementary file 1 — Supplementary file1 (DOCX 17 KB) [file 10126_2025_10500_MOESM1_ESM.docx]

**Table S1.** Ingredient composition (%) of the experimental aquafeed culture media for the extraction of ECP samples

|  | **F media** | **FM media** |
| --- | --- | --- |
| Fishmeal LT94 ^1^ | 10.0 | 10.0 |
| Soybean protein concentrate ^2^ | 15.0 | 9.2 |
| Wheat gluten ^3^ | 17.0 | 12.0 |
| Pea protein concentrate ^4^ | 5.0 | 5.0 |
| Soybean meal ^5^ | 20.0 | 12.0 |
| Wheat meal ^6^ | 14.14 | 9.94 |
| Fish oil ^7^ | 7.0 | 7.0 |
| Soybean oil ^8^ | 4.5 | 3.5 |
| Rapeseed oil ^9^ | 4.5 | 3.5 |
| Vitamin and mineral premix ^10^ | 1.0 | 1.0 |
| Vitamin C ^11^ | 0.05 | 0.05 |
| Vitamin E ^12^ | 0.01 | 0.01 |
| Methionine ^13^ | 0.5 | 0.5 |
| Monocalcium phosphate ^14^ | 1.3 | 1.3 |
| Microalgae ^15^ | 0 | 25.0 |

^1^ 69.4% crude protein, 12.3% crude lipid (Norsildemel, Bergen, Norway). ^2^ Soycomil, 60% crude protein, 1.5% crude lipid (ADM, Poland). ^3^ 78% crude protein (Lorca Nutrición Animal SA, Murcia, Spain). ^4^ Pea protein concentrate, 85% crude protein, 1.5% crude lipid (Emilio Peña SA, Spain). ^5^ Lorca Nutrición Animal SA (Murcia, Spain). ^6^ Local provider (Almería, Spain). ^7^ AF117DHA (Afamsa, Spain). ^8^ Soybean oil (Aceites el Niño, Spain). ^9^ Rapeseed oil (Roviroli, Barcelona, Spain). ^10^ *Lifebioencapsulation* SL (Almería, Spain). Vitamins (mg kg^-1^): vitamin A (retinyl acetate), 2,000,000 UI; vitamin D3 (DL-cholecalciferol), 200,000 UI; vitamin E (Lutavit E50), 10,000 mg; vitamin K3 (menadione sodium bisulphite), 2,500 mg; vitamin B1(thiamine hydrochloride), 3,000 mg; vitamin B2 (riboflavin), 3,000 mg; calcium pantothenate, 10,000 mg; nicotinic acid, 20,000 mg; vitamin B6 (pyridoxine hydrochloride), 2,000 mg; vitamin B9 (folic acid), 1,500 mg; vitamin B12 (cyanocobalamin), 10 mg vitamin H (biotin), 300 mg; inositol, 50,000 mg; betaine (Betafin S1), 50,000 mg. Minerals (mg kg-1): Co (cobalt carbonate), 65 mg; Cu (cupric sulphate), 900 mg; Fe (iron sulphate), 600 mg; I (potassium iodide), 50 mg; Mn (manganese oxide), 960 mg; Se (sodium selenite), 1 mg; Zn (zinc sulphate) 750 mg; Ca (calcium carbonate), 18.6%; (186,000 mg); KCl, 2.41%; (24,100 mg); NaCl, 4.0% (40,000 mg). ^11^ TECNOVIT, Spain. ^12, 23,14^ Lorca Nutrición Animal SA (Murcia, Spain). ^15^ Blend of *Chlorella fusca, Tisochrysis lutea, Microchloropsis gaditana,* and *Arthrospira platensis* (1.1:1:1) (LifeBioencapsulation, Almería, Spain)
